# MAGE-specific T cells detected directly ex-vivo correlate with complete remission in metastatic breast cancer patients after sequential immune-endocrine therapy

**DOI:** 10.1186/s40425-014-0032-2

**Published:** 2014-09-16

**Authors:** Maxwell Janosky, Rachel L Sabado, Crystal Cruz, Isabelita Vengco, Farah Hasan, Arthur Winer, Linda Moy, Sylvia Adams

**Affiliations:** NYU Laura and Isaac Perlmutter Cancer Center, New York University School of Medicine, 160 E 34th Street, New York, 10016 NY USA

## Abstract

Studies suggest that conventional cancer therapies given after immunotherapy (IT) can boost antitumor immunity and possibly improve response rates and progression-free survival. We report two cases of metastatic breast cancer with durable complete responses (CRs) after sequential IT and endocrine therapy. Immune analyses of these long-term disease-free breast cancer patients previously treated with imiquimod (IMQ) suggest in-situ vaccination is achieved by topical application of the TLR-7 agonist directly onto tumors. Furthermore, IT-induced antigen-specific T cells were expanded by subsequent endocrine therapy and correlated with response, persisting > 2 years. Our findings therefore suggest that the induction/boosting of polyfunctional tumor antigen-specific T in response to sequential immune endocrine therapy and detected directly ex-vivo can serve as a peripheral blood biomarker for true clinical benefit.

**Electronic supplementary material:**

The online version of this article (doi:10.1186/s40425-014-0032-2) contains supplementary material, which is available to authorized users.

## Introduction

Immunotherapy and harnessing anti-tumor immune effects of conventional cancer therapies have great potential for the treatment of several cancers. Evidence is emerging that subtypes of breast cancer can be immunogenic, and that the endogenous as well as the induced anti-tumor immune responses are linked to clinical outcomes in breast cancer [1]-[6]. However, the field has struggled to define immune monitoring benchmarks for clinical benefit. Several studies suggest that conventional cancer therapies given after immunotherapy, can further boost antitumor immunity and have shown higher than expected response rates and/or progression-free survival [7]-[12]. We report on two cases of metastatic breast cancer with extensive immune monitoring after an unusually favorable clinical course on sequential immunoendocrine therapy. Two of ten patients treated in a prospective study of topical imiquimod (IMQ), a synthetic imidazoquinoline and Toll-like receptor (TLR)-7 agonist, [13] applied to the involved skin overlying a large primary breast cancer had a complete and durable clinical response (CR) of all metastatic sites during subsequent therapy. We analyzed the longitudinal anti-tumor immunity in these two patients and demonstrate polyfunctional T cells specific to tumor and cancer testes (CT) antigens detected directly ex-vivo in the peripheral blood, coinciding with CR, suggesting a possible benchmark of beneficial immune modulation.

## Methods

### Patients

The prospective single arm study of imiquimod in metastatic breast cancer (clinicaltrials.gov NCT00899574, [13]) to assess response in skin metastasis was approved by the New York University IRB and was amended to collect follow-up blood in study patients. Patients consented to the research and photographs provided in this publication. Ten patients were enrolled and treated. IMQ was applied to cutaneous areas involved by tumor for 8 weeks. While their treated tumors did not regress on study, two patients entered a CR on the next line regimen with the antiestrogen fulvestrant. As CRs to fulvestrant are infrequent (1% in phase 3 trial) [14] and both are ongoing, we analyzed the patients' peripheral blood mononuclear cells (PBMCs) 2 years after enrollment into the IMQ trial. PBMCs were isolated from heparinized blood by Ficoll centrifugation and frozen in aliquots using pooled human serum (90%) and DMSO (10%).

### Intracellular cytokine analysis of ex-vivo PBMCs

PBMCs available from pre-IMQ-treatment, post-IMQ-treatment, and long-term follow up were thawed, washed in complete R-10 medium supplemented with 20 IU/ml DNase I (Roche) and cultured overnight at 37°C. The following day, 5 million viable cells/ml were placed in complete R-10 medium in the presence of 1 ug/ml anti-CD28 and anti-CD49d antibodies (BD Biosciences), and either 1 ug/ml PRAME and MAGE A3 overlapping peptides (Proimmune, 15mer peptides overlapping by 11 amino acids) or control antigens (ProMix CEF and MOG peptide pool; ProImmune). A mixture of Brefeldin A and Monensin (GolgiPlug and GolgiStop, BD Biosciences) was added after 1 hour culture, before culturing for an additional 5 hours. Samples were then washed with PBS, stained for 20 minutes at room temperature with anti-CD8 PerCP-Cy5.5 and anti-CD4 FITC antibodies (BD Biosciences) and LIVE/DEAD violet (Invitrogen), washed again with PBS, then fixed and permeabilized for 20 minutes at room temperature using Cytofix/Cytoperm solution (BD Biosciences). Samples were then washed using Perm/Wash solution (BD Biosciences) and stained for intracellular antigens using anti-IFN-γ AlexaFluor 700 (BioLegend), anti-TNF-α PE-Cy7, anti-IL-2 PE, anti-IL-4 APC, and anti-CD3 APC-H7 (BD Biosciences) antibodies for 20 minutes at room temperature. Samples were washed once with Perm/Wash solution, and acquired on a BD LSR II flow cytometer. 7-color compensation (parallel controls using cells singly stained for each color) and data analysis was performed with FlowJo flow cytometry analysis software (TreeStar). The induction or boosting of TAA-specific T cell immunity was defined as a post-treatment or long-term follow up value of at least 3-fold higher than baseline that is also 3-fold or greater than parallel negative controls (and at least 0.03).

**Intracellular cytokine staining (ICS) of peripheral lymphocytes after** ***in vitro*** **presensitization (IVS)** were performed by thawing PBMCs and culturing overnight in 10% PHS/RPMI, followed by separation into CD4+, CD8+, and CD4-CD8- (APC) fractions using Dynal Beads (Invitrogen). Purity of CD4+ and CD8+ fractions were 80-90% after separation. Each fraction was then washed and resuspended in 5% PHS/RPMI containing 10 U/ml IL-2 and 10 ng/ml IL-7. APCs were pulsed with ProMix/ProImmune peptide pool of the desired antigen (MAGE, PRAME, Her2, Survivin, or Wt-1, 5 μg/ml) for 1 h, irradiated (3000 rads) and then co-cultured with CD4+ or CD8+ cells (ratio of 1:1 or 1:2) in a 96-well round-bottom plate for 14-20 days, replenishing medium and cytokines every 2-3 days. After incubation, IVS T cell cultures were tested for reactivity to antigens (e.g. MAGE, PRAME) by ICS. IVS T cell cultures were harvested, washed, and replated in 10% PHS/RPMI medium in a 96-well V-bottom plate. Each test antigen peptide pool (1 μg/ml) was added to its own respective test well. Control wells containing DMSO, MOG, CEF, and PMA/Ionomycin were also included. For all ICS cultures, plates were incubated for 1 h at 37°C, after which BD GolgiPlug and GolgiStop was added to each well and the cultures incubated another 5 h. Cells were then stained for CD4 and CD8, fixed and permeabilized with BD Cytofix/Cytoperm solution, then washed with 1X BD Perm/Wash buffer and stained for CD3, CD4, CD8, IL-2, TNF-α, IL-4, IFN-γ and Live/Dead Violet. Cells were analyzed on a BD LSR II flow cytometer using FACSDiva software. Data were analyzed using FlowJo software (TreeStar).

## Results

**Figure 1:**
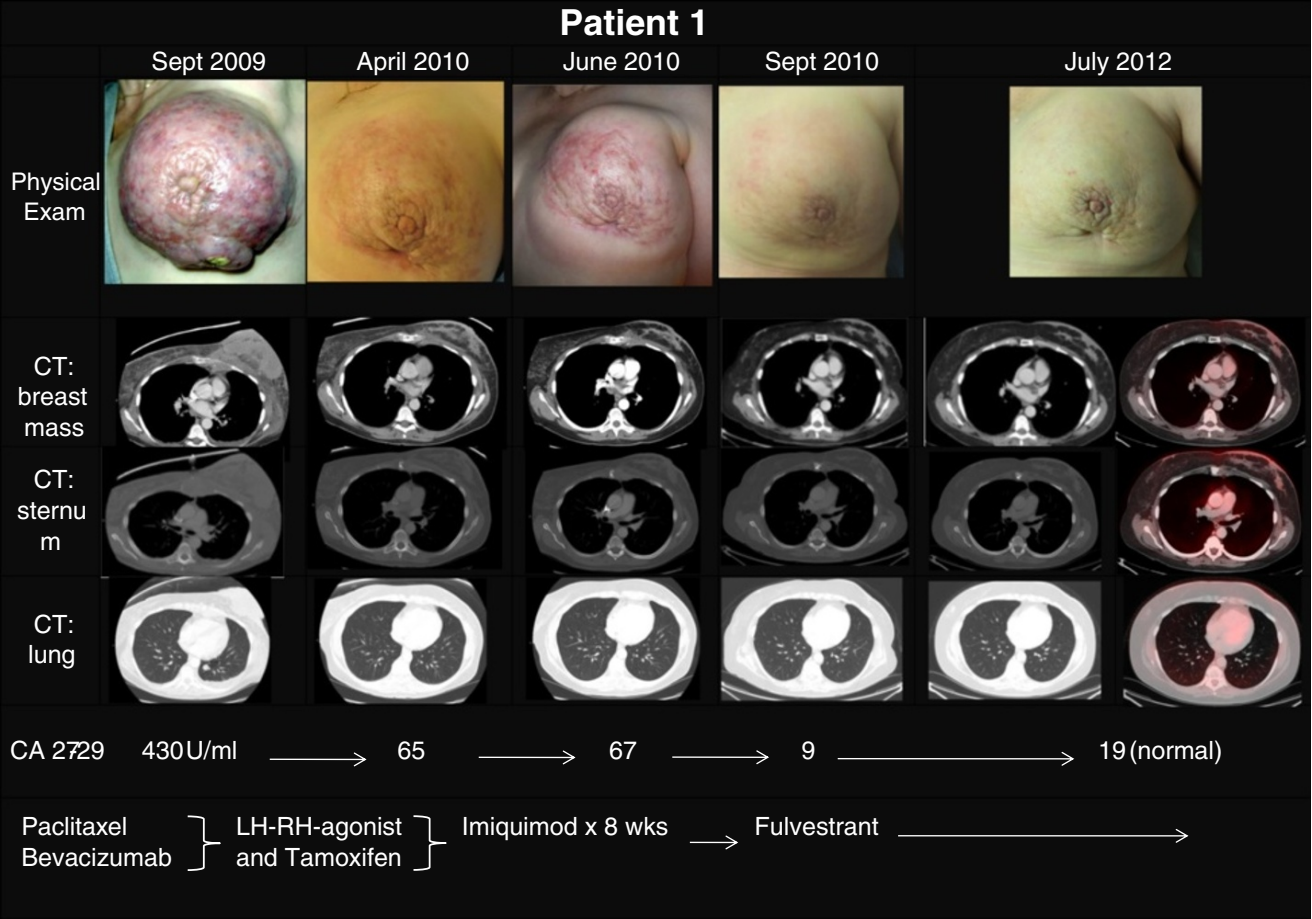
**Clinical exam photographs, imaging (CT scans and PET/CT available only at last follow-up), tumor marker (CA 27-29) and treatment regimens according to timeline for patient 1.** At initial presentation of the patient with malignant hypercalcemia in September 2009, the locally advanced breast cancer involved the overlying skin, invaded the pectoralis muscle and sternum; in addition lung metastases were detected by imaging as well as bone metastases (not shown). Interval treatments included paclitaxel, bevacizumab, LH-RH agonist, tamoxifen, topical imiquimod followed by fulvestrant, on which the patient experienced a complete response including breast, skin, lung and bone lesions in September of 2010. At last follow up August 2012, patient had no clinical or radiographic evidence of disease.

**Patient 1** is a 49 year old premenopausal woman who presented in September of 2009 at diagnosis with malignant hypercalcemia due to diffuse bone metastases of breast cancer. The primary tumor had replaced the entire left breast and invaded the skin, pectoralis muscle and sternum (Figure 1). Biopsy of the breast revealed a poorly differentiated, estrogen receptor (ER)-positive, human epidermal growth factor receptor (HER)-2 negative invasive ductal carcinoma; imaging tests confirmed widely metastatic disease with pulmonary and osseous metastases. After an initial course of chemotherapy with weekly paclitaxel and bevacizumab (in addition to bisphosphonates) which improved the patient's symptoms, treatment was switched to hormonal therapy with a LH-RH agonist and tamoxifen (with continued zoledronic acid). Upon progression 5 months later (April 2010), topical IMQ was added to all areas of involved skin. After one 8 week°Cycle the patient showed stable disease locally, however, imaging revealed one new lung metastasis, and the patient discontinued study treatment. Systemic therapy was switched to fulvestrant, on which the patient experienced a CR in September of 2010 including breast, skin, lung and bone lesions, which has been ongoing > 2 years (follow-up blood draws in February 2012 and April 2012).

**Figure 2:**
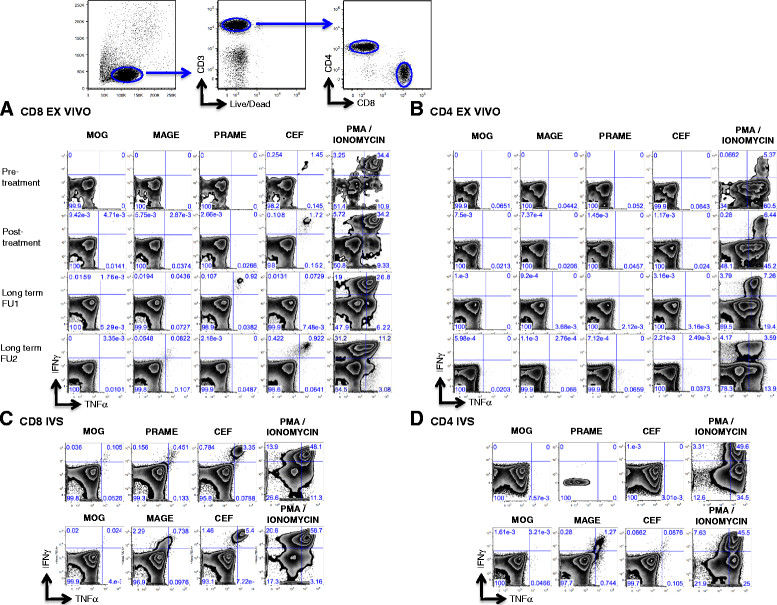
**Patient 1 antigen-specific responses pre and post IMQ treatment.** Ex-vivo MAGE and PRAME specific CD8+ **(A)** and CD4+ **(B)** T cell responses from bulk PBMCs of Patient 1 pre and post (post treatment and long term follow up 1 and 2) IMQ treatment were evaluated by intracellular cytokine staining for IFN-γ and TNF-α. CD8+ and CD4+ T cells were bead sorted from PBMCs collected post IMQ treatment and in vitro stimulated with CD8 and CD4 depleted PBMCs pulsed with MAGE or PRAME OLPs for 14-20 days. MAGE or PRAME specific CD8+ **(C)** and CD4+ **(D)** T cell responses were analyzed by intracellular cytokine staining for IFN-γ and TNF-α. Note that the flow plot for Prame looks different due to issues during acquisition of sample with too few cells. As controls, T cells were also stimulated with MOG and CEF OLPs and PMA/Ionomycin.

Immune monitoring results for patient 1 are shown in Figure 2. Before treatment with imiquimod, no MAGE or PRAME- specific T cells were detected in *ex-vivo* assays. After IMQ treatment, MAGE-specific T cells were detectable at very low frequency (0.04% of CD8+), mainly TNF-α secreting. Long-term PBMC evaluation (in the setting of complete clinical response on fulvestrant) revealed a sustained CD8+ T cell response detected directly ex-vivo to MAGE antigens (up to 0.189% of peripheral CD8+ lymphocytes), with a significant portion producing both, TNF-α and IFN-γ after antigen exposure. After IVS, MAGE-specific T cells showed significant expansion in the CD4+ and CD8+ compartments and confirmed polyfunctionality. MAGE-specific T cells did not secrete IL-4 or IL-2 (not shown). A CD8+ T cell response to PRAME was seen ex-vivo at LTFU1, but was only detectable after IVS by LTFU2. Pre-treatment samples were not available for IVS analysis.

**Figure 3:**
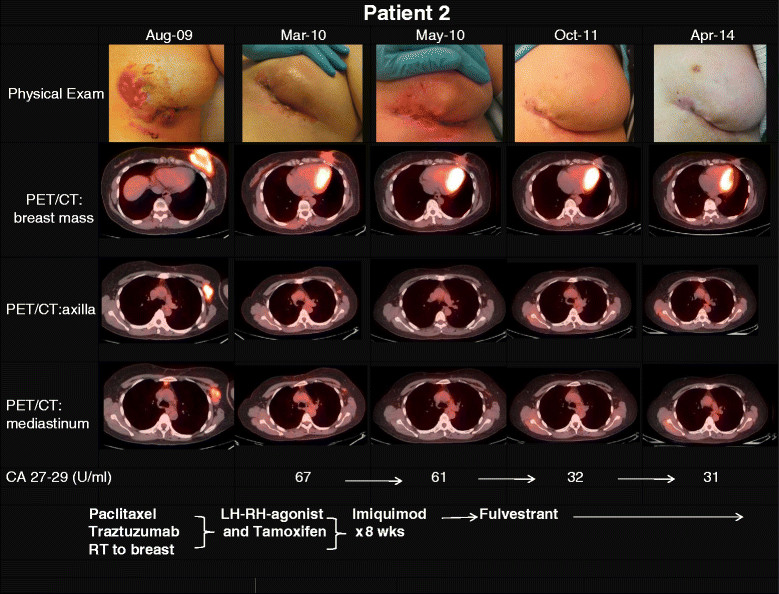
**Clinical exam photographs, imaging (PET/CT), tumor marker (CA 27-29) and treatment regimens according to timeline for patient 2.** At initial presentation of the patient in August 2009, the locally advanced breast cancer involved the overlying skin, axillary lymph nodes and invaded the pectoralis muscle. In addition an adrenal metastasis and mediastinal lymph nodes were detected by imaging. Interval treatments included paclitaxel, trastuzumab, LH-RH agonist, tamoxifen, topical imiquimod followed by fulvestrant, on which the patient experienced a complete response including breast, lymph nodes and adrenal lesions in October of 2011. At last follow up May 2014, patient had no clinical or radiographic evidence of disease.

**Patient 2** is a 50 year old premenopausal woman who presented in August 2009 with a fungating breast mass, involving skin and pectoralis muscle, which was histologically proven to be a poorly differentiated, ER- positive and HER2-positive invasive ductal carcinoma. Imaging demonstrated enlarged axillary and mediastinal lymph nodes as well as adrenal metastases (Figure 3). After initial therapy with trastuzumab and paclitaxel, including a course of concurrent radiotherapy to the breast for local palliation, taxane-induced neurotoxicity developed and treatment was switched to a LH-RH agonist and tamoxifen (with continued trastuzumab). Approximately 4 months later (March 2010), with stable disease, topical IMQ was added to all areas of involved skin. After one 8 week°Cycle the patient showed stable disease, however, she did not proceed to a second cycle. Upon disease progression 3 months later, systemic treatment was switched to fulvestrant (with continued trastuzumab), and she experienced a CR on this regimen in March of 2011, which has been ongoing >2 years. Follow-up bloods were obtained in April 2012.

**Figure 4:**
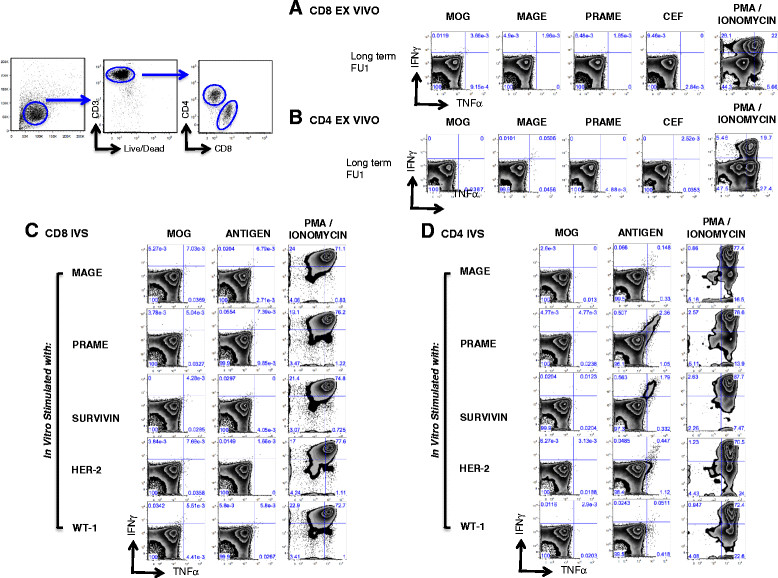
**Patient 2 antigen-specific responses post IMQ treatment.** Ex-vivo analysis of MAGE and PRAME specific CD8+ **(A)** and CD4+ **(B)** T cell responses from bulk PBMCs of Patient 2 by intracellular staining for IFN-γ and TNF-α. CD8+ and CD4+ T cells were bead sorted from PBMCs collected post IMQ treatment and in vitro stimulated with CD8 and CD4 depleted PBMCs pulsed with MAGE, PRAME, SURVIVIN, HER-2, or WT-1 OLPs for 14-21 days. Tumor antigen-specific CD8+ **(C)** and CD4+ **(D)** T cell responses were analyzed by intracellular staining for IFN-γ and TNF-α. As controls, T cells were also stimulated with MOG and CEF OLPs and PMA/Ionomycin.

Immune monitoring results for patient 2 are shown in Figure 4. As there was no functional recovery of PBMC after thawing for the pre- and post-IMQ time points, results are only available from long-term follow-up. MAGE-specific CD4+ T cells were detected *ex-vivo* (0.1%) during long-term follow-up (in complete clinical remission) (Figure 4B). IVS confirmed the presence of polyfunctional CD4+ MAGE-specific T cells, as well as reactivity to other tumor antigens PRAME, survivin, HER2 and Wt1 (not seen or tested for in *ex-vivo* assays) (Figure 4D). Antigen-specific T cells did not secrete IL-4 or IL-2 (not shown). No MAGE or PRAME specific CD8+ T cells were detected *ex-vivo* and after in vitro stimulation.

### Non-responder patients

**Figure 5:**
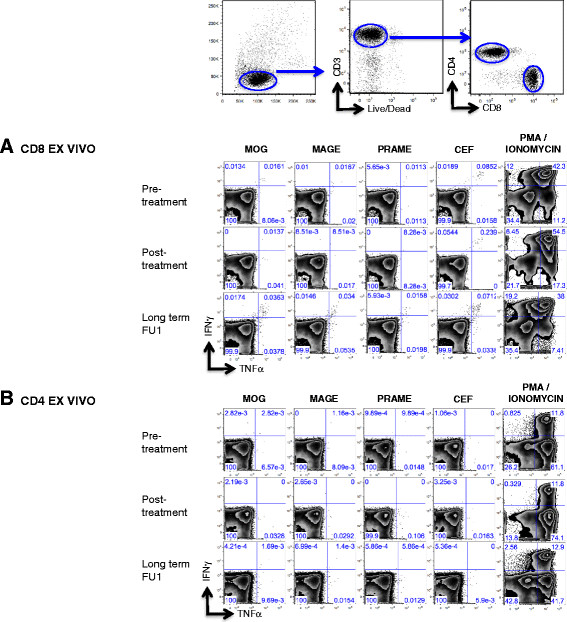
**Representative analysis of tumor antigen-specific responses in non-responder patient.** Representative ex-vivo analysis of antigen-specific CD8+ **(A)** and CD4+ **(B)** T cell responses from bulk PBMCs of one of three patients who also received IMQ treatment alongside Patient 1 and 2 by intracellular staining for IFN-γ and TNF-α. As controls, T cells were also stimulated with MOG and CEF overlapping peptides and PMA/Ionomycin.

Among the other eight patients who were treated on the IMQ trial, and progressed either on study or during subsequent therapies, three women who consented to follow-up blood draws died due to disease progression. In these three women, no immune response was detected to several tumor antigens including MAGE and PRAME by *ex-vivo* analysis; results of a representative patient are shown in Figure 5. Only for one of the three patients were paired samples available to additionally analyze the immune response by IVS. No CD4 or CD8 T cell response to MAGE was detected after IVS (data not shown).

## Discussion

We demonstrate that two patients with metastatic breast cancer who were treated with sequential immune-endocrine therapy and experienced durable complete responses had detectable antigen-specific T cells to several tumor antigens, in particular ex-vivo measurable MAGE A3-specific T cells. Importantly, these antigen-specific T cells were polyfunctional (IFN-γ+, TNF-α+, IL-4-, consistent with a Th1 profile) which has been associated with productive immune responses to vaccines [15]. Parallel evaluation of PBMCs of one patient from before and after IMQ treatment revealed that, while not detectable at baseline, IMQ treatment of the primary tumor involving the skin induced low frequency MAGE-A3-specific CD8+ T cells which were significantly expanded during subsequent endocrine therapy. In contrast, three patients who died due to subsequent progression did not have detectable MAGE A3-specific T cells.

Although IMQ can induce both CD4 and CD8 T cell responses [16],[17], the induction of CD4 versus CD8 T cell responses in these two patients may be partly attributable to the differential effects of prior chemotherapy/bevacizumab on the function of antigen presenting cells [18] as well as differences in the complex tumor microenvironment [19]. Regardless, both CD4 and CD8 T cell responses are necessary for induction of productive immune response against tumors [20].

Long-term durable responses are rare in metastatic cancer. However, immunotherapy trials followed by chemo- or endocrine therapy have demonstrated in some patients an unexpectedly high response rates and prolonged progression-free survival [7]-[9]. Patients who respond to immunotherapy or sequential therapies rarely have simultaneous immune monitoring to define the magnitude of immune response boost, correlatives of response and what differentiates the responders from the non-responders, studies which are crucial to the development of more effective therapies. One trial of metastatic breast cancer patients demonstrated an association of the presence of IFN-γ + tumor antigen-reactive peripheral T cells with long-term survival after adoptive transfer of antigen-specific T cells [21]. In this study, even with *in vitro* reactivation of tumor antigen-specific T cells derived from patient bone marrow only 1.7% of PMBCs were antigen specific CD8 cells [22]. Vaccine studies have typically induced lower frequencies of antigen-specific T cells. HER2 vaccination with three peptides led to a level of 1 HER2-specific T cell to 6,000 PBMCs [23]. In another HER2 vaccine study, at baseline 0.38% of CD8 cells were HER2-specific. With serial vaccination, the maximum expansion reached 1.8%, but levels returned to 0.45% at 6 months (not statistically significantly increased from baseline) [24].

In addition to HER2 and PRAME which are expressed in 17% and 53% of breast cancers, respectively [25],[26], CT antigens have also been targets for vaccine trials and are desirable antigens due to their restricted expression pattern [27]. Several cancer vaccine trials of CT antigens such as MAGE-A3 and NY-ESO-1 have demonstrated the ability to induce cellular and humoral immune responses, especially when used with potent vaccine adjuvants including TLR agonists [28],[29]. The persistence of vaccine-induced B and T cell memory responses years after booster immunization has also been demonstrated [28]. Consequently, the National Cancer Institute has placed two CT antigens, MAGE-A3 and NY-ESO-1, into the top 10 category of the Project for the Prioritization of Cancer Antigens [30].

While studies have reported varying frequencies of CT antigen expression in primary breast cancer [31]-[41], expression data are rarely available for paired samples from the same patient experiencing distant metastases. In melanoma and lung cancer, studies from our group and others reported a higher expression of CT antigens in metastatic deposits compared with primary lesions, which suggests that these antigens are frequently acquired during progression [42],[43]. While robust expression data for breast cancer in primary tumors and subsequent metastases are not available, a study using multiple MAGE-recognizing primers (MMRPs) that can simultaneously detect 6 MAGE-A gene subtypes (MAGE-A1-MAGE-A6) expressed by circulating tumor cells in patients with breast cancer suggests that increasing MAGE-A gene expression predicts for tumor progression or recurrence [44].

Our immune evaluation of long-term disease-free breast cancer patients suggests in-situ vaccination is achieved by application of the TLR7- agonist directly onto tumors and subsequent endocrine therapy can expand these responses. Furthermore, we suggest that antigen-specific immune responses detected directly ex-vivo during freedom of detectable disease may indicate a contribution of the immune response to disease control in metastatic breast cancer. This is particularly plausible as the cytokine profile of these cells is similar to T cells specific to infectious agents including CMV-responsive T cells, and unlike the profile seen in breast cancer where the majority of preexisting cancer antigen T cells fails to produce IFN-y [45].

Strengths of this study include the availability of cryopreserved PBMCs from various time points, which allowed an immune analysis from two breast cancer patients with rare durable responses, and treatment with IMQ on a clinical trial with prospective follow up for 2 years and the willingness of the two long-term responders as well as several patients with disease progression (controls) to donate blood samples. Antigens for immune monitoring were chosen based on their use in vaccine trials and expression in breast cancer. Limitations of our study are that we were unable to test the patients' tumor expression for MAGE and PRAME, although HER2 expression was demonstrated by standard immunohistochemistry in the primary tumor of the patient who developed anti-HER2 T cell responses. MAGE-A3 antibodies were not detected in any of the patients at any time point (data not shown), HER2 and PRAME antibodies were not measured. Furthermore, due to limited samples we could not evaluate the anti-tumor cytotoxicity of these T cells *in vitro*. In addition, the lack of the pre-treatment samples for patient 2 limits the interpretation of our findings.

## Conclusion

In summary, we demonstrate that IT-induced antigen-specific T cells can be expanded by subsequent endocrine therapy, co-secrete IFN-γ and TNF-α and correlated with complete and durable responses. This suggests that the induction and boosting of TAA-specific T cells detected directly ex-vivo is a benchmark for true clinical benefit in patients with metastatic breast cancer. Therefore, biomarkers in the blood may be appropriate surrogates for predicting clinical responsiveness in the future.

## Authors’ original submitted files for images

Below are the links to the authors’ original submitted files for images. Authors’ original file for figure 1
Authors’ original file for figure 2
Authors’ original file for figure 3
Authors’ original file for figure 4
Authors’ original file for figure 5
