# Supplementary figures and images for: MAGE-specific T cells detected directly ex-vivo correlate with complete remission in metastatic breast cancer patients after sequential immune-endocrine therapy
Source: J Immunother Cancer. 2014 Sep 16;2:32. doi: 10.1186/s40425-014-0032-2 (PMC5569937; doi:10.1186/s40425-014-0032-2)

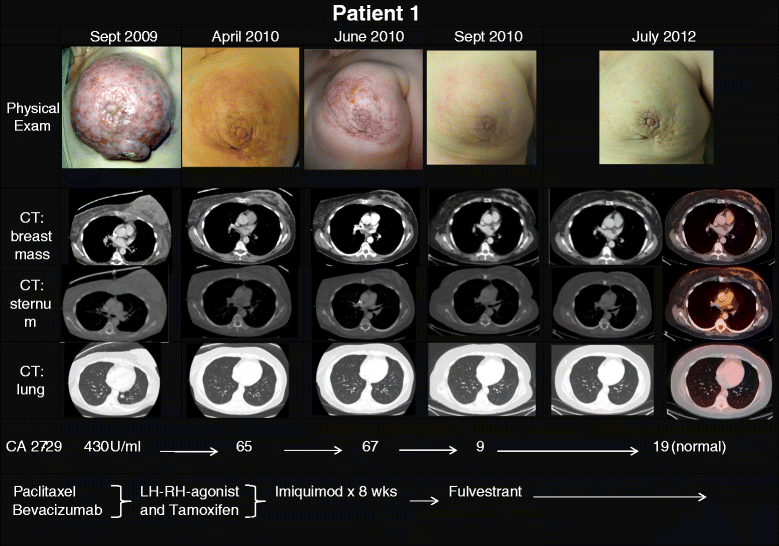

Supplement: Supplementary file 1 — Authors’ original file for figure 1 [file 40425_2014_32_MOESM1_ESM.gif]

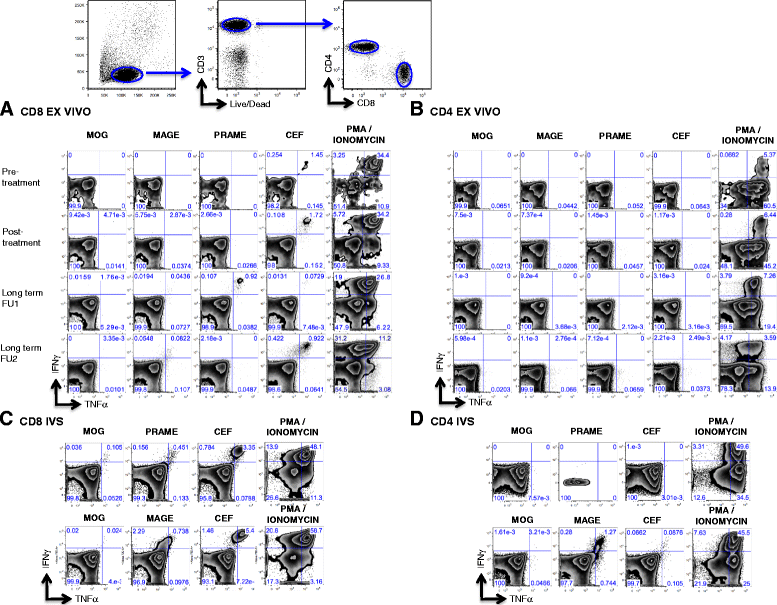

Supplement: Supplementary file 2 — Authors’ original file for figure 2 [file 40425_2014_32_MOESM2_ESM.gif]

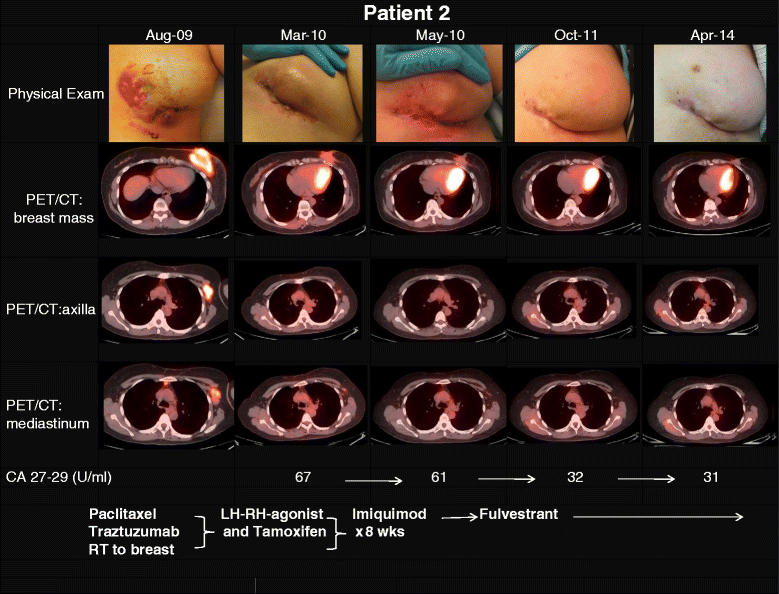

Supplement: Supplementary file 3 — Authors’ original file for figure 3 [file 40425_2014_32_MOESM3_ESM.gif]

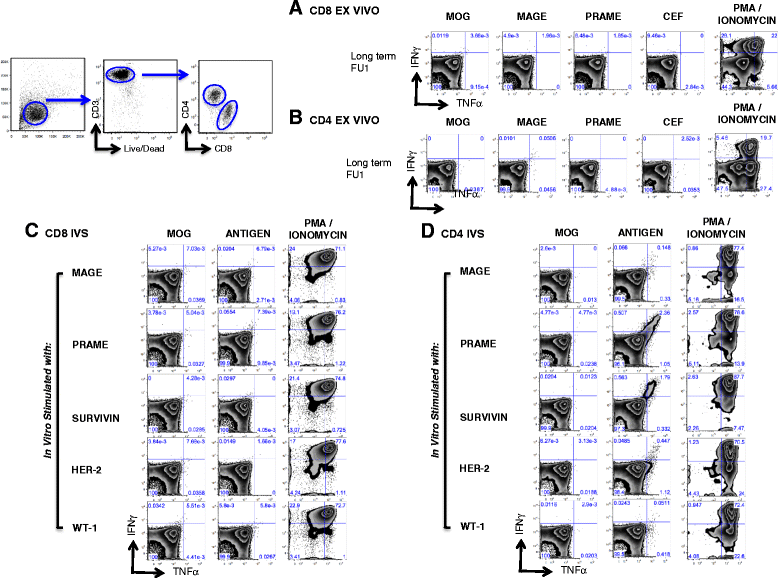

Supplement: Supplementary file 4 — Authors’ original file for figure 4 [file 40425_2014_32_MOESM4_ESM.gif]

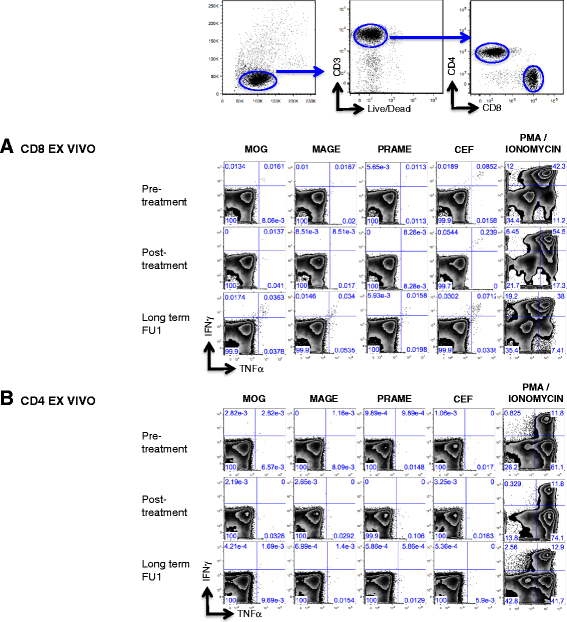

Supplement: Supplementary file 5 — Authors’ original file for figure 5 [file 40425_2014_32_MOESM5_ESM.gif]
